# Making Sense of Biodiversity: The Affordances of Systems Ecology

**DOI:** 10.3389/fpsyg.2018.00594

**Published:** 2018-05-04

**Authors:** Erik Andersson, Timon McPhearson

**Affiliations:** ^1^Stockholm Resilience Centre, Stockholm University, Stockholm, Sweden; ^2^Urban Systems Lab, The New School, New York, NY, United States; ^3^Cary Institute of Ecosystem Studies, Millbrook, NY, United States

**Keywords:** functional traits, reciprocal interactions, ecosystem function, ecosystem services, biodiversity, affordances

## Abstract

We see two related, but not well-linked fields that together could help us better understand biodiversity and how it, over time, provides benefits to people. The affordances approach in environmental psychology offers a way to understand our perceptual appraisal of landscapes and biodiversity and, to some extent, intentional choice or behavior, i.e., a way of relating the individual to the system s/he/it lives in. In the field of ecology, organism-specific functional traits are similarly understood as the physiological and behavioral characteristics of an organism that informs the way it interacts with its surroundings. Here, we review the often overlooked role of traits in the provisioning of ecosystem services as a potential bridge between affordance theory and applied systems ecology. We propose that many traits can be understood as the basis for the affordances offered by biodiversity, and that they offer a more fruitful way to discuss human–biodiversity relations than do the taxonomic information most often used. Moreover, as emerging transdisciplinary studies indicate, connecting affordances to functional traits allows us to ask questions about the temporal and two-way nature of affordances and perhaps most importantly, can serve as a starting point for more fully bridging the fields of ecology and environmental psychology with respect to how we understand human–biodiversity relationships.

## Introduction

### Biodiversity and Human Affordances

Biodiversity provides the principal basis for ecosystem services important for human life and well-being (Cardinale et al., 2012; Bennett et al., 2015). Despite substantial scholarly progress, we do not adequately understand the relationship between different types and levels of ecosystem, functional, or species diversity and the many aspects of human well-being, whether they are in agricultural, urban, or other landscape contexts (McPhearson et al., 2016; Bennett, 2017). At the same time, there is growing awareness and consensus that human-induced biodiversity loss is elevating socioeconomic risks and costs, undermining human well-being, and the unique opportunities afforded by ecosystems (Steffen et al., 2015; Ouyang et al., 2016).

We need to better understand (1) biodiversity and how it contributes to human wellbeing, (2) how human use in turn influences biodiversity, and (3) pathways for pro-environmental behavior. *Affordance theory*, based on extensive scholarly work in environmental psychology (Chemero, 2009; Kaaronen, 2017), provides a systemic framework for analyzing interactions as relational and situation specific outcomes. Affordances are defined in this paper as the “relations between *abilities* to perceive and act and *features* of the environment” (Chemero, 2009, p. 252, our emphasis), and we follow Chemero (2003, 2009) in that we take affordances to mean functionally meaningful whole situations. Interactions with species and ecosystems have the potential to support or afford multiple human well-being outcomes (Díaz et al., 2018). However, human–biodiversity interactions are reciprocal and the biodiversity response to human activity (i.e., human-driven species loss) will eventually influence which affordances will be available in the future (Chapin et al., 2000). The existing environmental psychology literature recognizes the role of ecosystems, but, we argue, does not adequately capture enough ecological detail to influence the management of these “features of the environment” for improved human health and well-being, nor for making sure these opportunities are resilient over time in the face of local and global environmental change.

### Indirect Effects and Time Dynamics

Ecosystems are not static, nor are they a single entity that humans interact with. Rather, ecosystems have species, processes, and functions, all of which are both acting upon each other and reacting to abiotic and biotic change. As we interact with our surroundings, we change (and are changed by) them, and many of the environmental problems we face are results of careless or deliberate exploitation of opportunity. For example, policy and management decisions in the past could have been made to maintain sustainable supply of fish in many regional fisheries over time, thus requiring limits on the amount of fish caught. However, what is much more common is the rampant exploitation to the point of depletion of fish stocks in fisheries all over the world that has driven global fish stocks near ecological collapse (e.g., Pinsky et al., 2011). Digging deeper into the ecological outcomes of human–biodiversity relationships is critical if we are to understand them well enough to improve both ecosystem health and the ecological contribution to human well-being. While recognized in affordance theory, this ecological side of dynamic interactions is explored more in depth in ecology.

In this paper, we describe the role of *functional traits* in ecosystem functioning and for human affordances, and explore the concept’s potential to further bridge the fields of ecology and environmental psychology. Below, we outline some of the central insights and considerations from the functional traits literature, especially where it has engaged with human perceptions and values. We then use *sense of place* and *focal species* to illustrate ongoing research where elements of affordance theory and ecology are already now used together to better capture the dynamics of social–ecological systems. Finally, we build on these to identify some of the most promising areas where a joint research agenda could support sustained ecological integrity together with diverse human affordances.

## Functional Traits for Understanding System Dynamics and Affordances

### What Biodiversity Affords

Essentially, ecological functions and dynamics over time are mediated by biodiversity and complex interactions between organisms and their surroundings (Chapin et al., 1997; Norberg, 1999). Organisms have an effect on the environment they live in by creating or contributing certain attributes, abilities, and opportunities for interactions, which may serve as the basis for ecosystem services and thus human affordances (Díaz and Cabido, 2001; de Bello et al., 2010; Stokols, 2017). Early studies trying to connect biodiversity to ecosystem dynamics and function used species richness (the number of different species in a community), with some success (e.g., Tilman et al., 1997; Tilman, 1999). However, taxonomic biodiversity has since been criticized as being a blunt analytical framework for describing and understanding species interactions and their outcomes (Mori et al., 2013). Multiple studies (e.g., Díaz and Cabido, 2001; Cornelissen et al., 2003; Vandewalle et al., 2010; McDonnell and Hahs, 2013) suggest instead that functional traits – those abilities and features of organisms with demonstrable links to its ecosystem role and performance and, in turn, fitness – may provide a useful and more mechanistically informative alternative. The approach has been adopted historically for descriptive reasons (McDonnell and Hahs, 2013), to enable broader global comparisons that transcend the constraints placed on such studies by regional taxonomic diversity, and allow for the types of generalizations (e.g., responses to environmental change, ecological implications of trends and patterns) sought after in ecology (Cornelissen et al., 2003; Blaum et al., 2011).

Affordance theory and ecology share many meta-theoretical components: Both view the environment as produced through ongoing, adaptive interactions (e.g., trophic interactions), between organisms (humans included) mediated through abilities and features set in time-specific situational contexts (Pickett et al., 2005; Heft, 2013). And while some traits-based work still uses traits primarily as functional attributes of discrete objects (e.g., comparative studies), especially studies of traits connected to ecosystem services (Díaz et al., 2007; de Bello et al., 2010; Lavorel et al., 2011) have taken on an organismic or even (if to a lesser degree) transactional ontological stance (*sensu* Altman and Rogoff, 1987). Similar to affordances, functional traits describe how and why an organism interacts with its surroundings, capturing and detailing both abilities and features. For example, birds have been grouped and described according to factors such as beak shape, wing length, migratory status, territorial behavior, diet, and foraging strategies (e.g., Simberloff and Dayan, 1991). These factors have a direct bearing on how the ecosystem functions (Sekercioglu et al., 2004) and may serve as mediators of interactive change (e.g., seed dispersal and competition).

The recent expansion of traits-based work to include a more explicit treatment of human views, values, and perspectives (Goodness et al., 2016) and discuss how these demand new traits to be added to the developing trait lists. Díaz et al. (2011) have described a number of tools now available to quantify functional diversity and link it with ecosystem properties and services. For example, the literature contains a growing evidence based on functional traits that influence ecosystem properties in predictable ways (Lavorel and Garnier, 2002; Cornelissen et al., 2003). These traits include leaf size and chemical composition, seed size and longevity, and canopy and root architecture which affect the ability of a plant species to establish, grow quickly, be productive, reproduce, and respond to disturbances. Standardized low-tech protocols are available for the measurement of these traits (Cornelissen et al., 2003) and the number of metrics for the quantification of traits are growing quickly (Villéger et al., 2008).

We can then use the functional traits approach to better understand which affordances a specific setting may offer and what ecosystem services may or may not exist. The diversity of traits opens up possibilities and multiple species with similar traits offer potential redundancy in the ecological set-up and support of different affordances.

### Feedback and Temporal Dynamics

**FIGURE 1:**
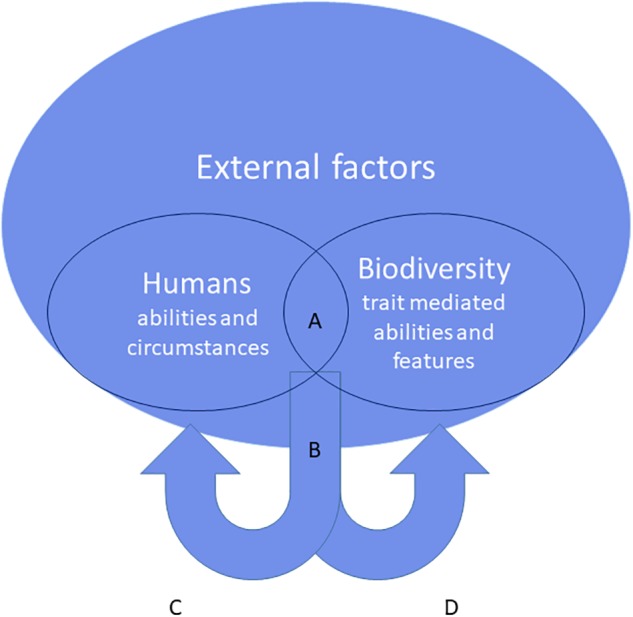
The reciprocal interaction between people and biodiversity. Through their features and abilities (mediated by functional traits) organisms create opportunities for affordances **(A)**. These become real when they are complemented by human abilities and external factors. Realization of affordances **(B)** through an interaction between the organism and a person may ultimately confer direct and indirect benefits for human health and well-being **(C)**. The response of the organism to the same interaction is again mediated by functional traits **(D)**. The interaction and its outcomes may change the future availability of the initial set of affordances.

At the landscape scale, where most of our actions play out, knowledge on traits could inform management and planning so that it effectively improves the functioning and resilience of the ecological palette upon which human well-being is dependent (e.g., Díaz et al., 2011). Perception–action processes are not static; they happen over time and their actualization changes the subsequent patterns of relations between humans and biodiversity (Chemero, 2009), and reciprocal effects can potentially affect the future opportunities to have the same type of interaction (**Figure 1**). Researchers have documented how people consciously and unconsciously protect, conserve, use, contest, alter, exploit, destroy, change, and rehabilitate ecosystems, either for their own or someone else’s benefit, and all such actions have implications for ecosystem functions and services. At present, there is little systematic understanding about the particular combinations of different human actions and different ecological systems that provide ecosystem services and even less knowledge about service provisioning that is sustainable, efficient, and equitably now and in the future (Bennett et al., 2015). The functional traits approach can help use disentangle such feedback mechanisms (Tomimatsu et al., 2013). The nature of direct human–biodiversity interaction (frequency, intensity, and timing all matter) offers a way to assess its relative strength compared to the abiotic (climatic, resource availability, disturbance) and biotic (competition, predation, mutualisms) factors that over time influence the local pool of species and traits (reviewed in Diaz et al., 1999). Lavorel and Garnier (2002) argue that we should be able to predict the trait pool of a species community by combining the knowledge of the nature and strength of different factors with the trait-mediated response to each factor.

By having redundant options among suites of species with particular traits, we have choices, and stand a better chance that the affordance desired will remain present in the system over time and have the necessary traits or diversity of traits to be resilient to disturbance or stress (Walker, 1992). If the different species offering the same affordance differ in their responses to external factors, it is more likely that one will have a diversity of responses to any stress or change that the system it is exposed to (Mori et al., 2013). For example, urban trees that can tolerate air pollution, drought, and soil compaction are more likely to persist in urban environments, for example as street trees, thus providing opportunity for affordances in ways trees without these traits may not offer the same opportunities. This response diversity is the most direct linkage between biodiversity and the overtime resilience of system function and structure (Elmqvist et al., 2003), and is thus a critically important prerequisite for making sure affordances remain in the system.

## Co-Production and Sense Making

As recognized in affordance theory, nuances in meaning and the range of affordances offered by any ecosystem are connected to ecological attributes at different levels, from landscapes down to genes (Stokols, 2017), and current ecological communities are products of social–ecological dynamics. In addition to offering direct affordances, the biophysical environment also provide one of the foundations for registering and conceptualizing change. There are several examples in transdisciplinary studies where differences in ecological character (which are often made up by suites of traits) have been coupled with external as well as internal human factors. We have chosen two of these to serve as illustrations of the questions we can start asking by combining in-depth disciplinary knowledge and approaches.

### Sense of Place

Research on the connections between people and places has sought to capture how emotional, experiential and cultural connections mediate human perception and response to change, among other things. Place meanings were initially understood as primarily social constructs, a view that was challenged by Stedman (2003), who argued that the biophysical world imposes both clear constraints and opportunities for creating different meanings. Following this line of understanding, sense of place can be said to capture both the attachment to place and the qualities and descriptive meanings one is attached to (Masterson et al., 2017). Scholars have distinguished between place attachment and place meaning (Scannell and Gifford, 2010), emphasizing the different meanings and affordances place has to the people directly or indirectly connected to it. Where place attachment focuses on our emotional bond, place dependence and reciprocally informed identity, place meaning emphasizes the descriptive cognitive description of what a place is and what meanings it holds. Both are clearly the results of multiple internal and external factors, and especially place identity and place meaning are strongly influenced by the character of the biophysical setting (Stedman, 2002; Masterson et al., 2017). The sense of place literature has also shown how perceived changes in available affordances (often with specific traits serving as cues) can serve as triggers for direct action grounded in a deep sense of care and responsibility (Enqvist, 2017). This idea of “cues” or “triggers” has also been taken up by conservation biology as well as conservation psychology (e.g., Charles Vlek, 2007; Gifford, 2007; Chapin et al., 2012).

### Conservation Biology and the Use of Focal Species

Conservation biology has long used individual species as foci for different discussions and campaigns, for example pandas or polar bears. The choice of species is based on different attributes that are seen as mediators of meaning beyond the biological organism itself. This literature offers both clear evidence of the value of combining psychological, social, and ecological aspects, and the challenges amidst this complexity. From an ecological integrity perspective, the focus has been on species that have a particularly significant impact on the state of a community or an ecosystem, either based on significant life history traits or niche (e.g., keystone predators), or because the species is highly interactive and abundant (Soule et al., 2003). Alternatively, when less is known about ecological relationships, species with relatively extensive habitat requirements serve as “umbrellas” for conservation (Simberloff, 1998). The choice of focal species may also be grounded in traits that relate to its appearance, charisma, behavior and utility (Walpole and Leader-Williams, 2002; Serpell, 2004; Lorimer, 2007; Martín-López et al., 2008), characteristics that relate to the importance of affect as a vital motivating force for people to get involved in conservation efforts (Lorimer, 2007).

This approach to conservation suggests that efforts should focus on key endangered interactions between species (humans included), not just on endangered species. Further, we suggest that the different meanings and different traits can be combined to capture biodiversity responses to various interactions with people, cascading effects of ecological change, as well as what the consequences might be for people. Kronenberg et al. (2017) suggested that a “social-ecological keystone species is likely to be more meaningful for broader conservation objectives because it complements the ecological importance with the social perception of a species, thereby opening an opportunity to connect various dimensions of social/cultural value that people attribute to nature to ecological quality and dynamics.” Similar to sense of place, this combination of extended biophysical and socio-cultural meanings and relations highlights our own role as stewards, framing conservation as not only needed to preserve species and ecosystems, but because we impact the different avenues for meaningful human interactions with these ecological components. In the language of this article, this means that recognizable, legible functional traits are important to use (analytically as well as actively) to understand and support pro-environmental affordances.

## Conclusion: What is Biodiversity to us? An Open Invitation to Join in the Exploration of Meaningful Interactions

There is much work remaining to integrate the affordances perspective with research on ecosystem dynamics and ecosystem services (Raymond et al., 2017). In this mini-review we have presented functional traits as a bridge for connecting affordances to biodiversity and the real dynamics of ecosystems, and thus add an in-depth ecological perspective to the environmental psychology field and the complex topic of human–environment interactions. There are two areas in particular where we see a clear benefit in trying to combine the two frameworks. First, biodiversity studies have long grappled with the cultural understanding and making sense of biodiversity, and insights from environmental psychology may help us understand why and under what circumstances an opportunity leads to interaction and the human ability to process sensory input. Intrinsic, deeply embedded, and culturally framed meanings have long been recognized for ecosystems in general, but the ties with biodiversity remain tenuous. Second, for environmental psychology to have impact on management, design, or planning to improve the functions and services of ecosystems, it needs improved ecological specificity, which can be helped with a traits-focused approach.

Both the reasons for and the benefits of a certain human action – the realization of an affordance – are complex bundles of contingencies and the nature of reciprocal interaction is important. The theory of affordances stem from an ontology where the nature of the interaction is as important as the underlying factors that constitute an affordance (Gibson and James, 1979), and ecosystem services are increasingly seen as co-produced (e.g., Andersson et al., 2015; Palomo et al., 2016). However, this understanding has yet to strongly connect to the functional traits studies. An affordance is dependent on not just one detail of the system (e.g., species or conceptualization of an ecosystem, or presence of a specific trait), but on a number of factors, some internal, some external, some ecological, and some socio-cultural. Kyttä (2004) pointed to the need to better understand and account for the full suite of factors influencing whether or not an affordance is realized. Once an individual has perceived an opportunity for action afforded by the environment, the actual realization only emerges when the different characteristics of the individual, such as his or her physical abilities, social needs, and personal intentions, align with and match the opportunity space (Kaaronen, 2017). In addition to the embodied perspective offered by affordance theory, there are layers of sense making and social constructs like institutional regulations and norms that all influence which affordances are recognized and how they are realized, and thereby what the implications are for the biodiversity involved in the interaction.

By harnessing the dual aspects of functional traits, we can better understand the implications of people responding to desired or attractive traits. The attractive trait expression (perceived at any level of biodiversity, genes, species, communities, and ecosystems) comes with sets of individual species response traits that will inform both how an organism may respond to the realization of an affordance and how sensitive different affordances are to larger-scale environmental changes. Additionally, the ecological literature tells us that there are alternative biodiversity configurations that may offer the same affordances. The affordance literature in turn reinforce that there are numerous ways in which an affordance can be realized. Affordances are only *opportunities* for action; the outcomes are complex and not necessarily that well-recognized beyond the direct experience of the interaction and final service provided by the dynamics of the environment. Many of the indirect and less desirable outcomes remain less obvious.

Linking environmental psychology and systems ecology together can help us:

– Bridge the scales of human perception and the often more detailed information about species traits;
– Understand when, why, and how an affordance is realized;
– Seek redundancy and resilience in the functions of environments by establishing redundant affordances that inspire interaction pathways with low negative impact;
– Explore indirect benefits, e.g., pro-environmental behavior of biologically diverse everyday landscapes;
– Co-produce environmentally friendly affordances by combining biodiversity with design and sense-making.

Advancing such research requires interdisciplinary collaboration and deepening the understanding of complex dynamics in ecosystems, but also new data sources to build more extensive and relevant trait databases to advance the linkages between systems ecology and environmental psychology.

## Author Contributions

Both authors have substantially contributed to the conception or design of the work and provided critically important intellectual content. The outline and design of the study was led by EA. Both authors approved the submitted version of the manuscript and both agreed to be accountable for all aspects of the work in ensuring that questions related to the accuracy or integrity of any part of the work are appropriately investigated and resolved.

## Conflict of Interest Statement

The authors declare that the research was conducted in the absence of any commercial or financial relationships that could be construed as a potential conflict of interest.
